# Has the Risk of Outpatient Visits for Allergic Rhinitis, Related to Short-Term Exposure to Air Pollution, Changed over the Past Years in Beijing, China?

**DOI:** 10.3390/ijerph191912529

**Published:** 2022-10-01

**Authors:** Sai Li, Gang Wang, Beibei Wang, Suzhen Cao, Kai Zhang, Xiaoli Duan, Wei Wu

**Affiliations:** 1 School of Energy and Environmental Engineering, University of Science and Technology Beijing, Beijing 100083, China; 2 Department of Otolaryngology-Head and Neck Surgery, PLA Strategic Support Force Characteristic Medical Center, Beijing 100101, China; 3 Department of Environmental Health Sciences, School of Public Health, University at Albany, Rensselaer, NY 12144-2345, USA

**Keywords:** air pollution, temporal variation, allergic rhinitis, outpatient visit

## Abstract

A number of studies have found associations between the short-term exposure to ambient air pollution and hospital admissions. However, little is known about the temporal variations in ambient air pollution associated with health exposure, especially in China. We evaluated whether the risks of allergic rhinitis (AR) outpatient visits from short-term exposure to air pollution varied over time (2014–2020) in Beijing, China. A quasi-Poisson generalized additive model was used to evaluate the relative risks (RRs) and 95% confidence intervals (CIs) associated with the pollutant concentrations during the entire study period and three specific periods. We also analyzed the temporal variations of the period-specific associations and tested the trend of change using the Mann–Kendall test. The concentration-response relationships for the specific periods were further investigated. The RRs (95%CI) for an interquartile range (IQR) increased in PM_10_ (70 μg/m^3^) and CO (0.5 mg/m^3^) decreased from period 1 to period 3. However, The RRs (95%CI) of PM_2.5_ (55 μg/m^3^), SO_2_ (7 μg/m^3^) and NO_2_ (27 μg/m^3^) increased from 1.015 (0.978, 1.054), 1.027 (1.009, 1.044) and 1.086 (1.037, 1.137) in period 1 to 1.069 (1.005, 1.135), 1.074 (1.003, 1.149) and 1.214 (1.149, 1.282) in period 3, respectively. A statistically significant temporal change and the stable effects were observed between the NO_2_ exposure and AR visits over time. Despite a substantial reduction in ambient air pollution, the short-term effects on AR outpatient visits remained significant. Our findings provide a rationale for continued air pollution control efforts in the future to minimize air pollution and to protect the public.

## 1. Introduction

Air pollution has been considered a serious threat to public health worldwide. The impact of air pollution on health has attracted increasing attention and has been reported in a variety of countries, considering cardiovascular and respiratory diseases, neonatal conditions and hospital admissions [1,2,3,4]. However, most of the studies focused on the overall effect during the study period. The epidemiological evidence on the temporal changes in the health effects of air pollution is still limited [5,6,7,8]. Previous studies have suggested that there may be temporal differences in the health effects of air pollutants, but the available research evidence remains inconsistent.

In response to economic development and urbanization, the Chinese government has developed a series of air pollution control measures and policies nationwide to improve air quality. Beijing and neighboring areas are among the most stringent target areas. In most cities in China, the concentration and composition of air pollutants have varied considerably with time. During the past few years, concentrations of sulfur dioxide (SO_2_) and particulate matter ≤ 2.5 μm in diameter (PM_2.5_) and ≤10 μm (PM_10_) have declined in most Chinese cities [9,10]. However, the annual average concentration of PM_2.5_ in Beijing still exceeds the Chinese national ambient air quality standard (NAAQS) of 35 μg/m^3^ for PM_2.5_, and traffic pollution is still serious. In addition to this, air pollution restrictions may also change the precise emission sources as well as the chemical composition of the air pollution mixture. The emissions from the chemical industry, manufacturing and transportation-related air pollutants may be reduced, which are closely related to the emission concentrations of particulate matter, SO_2_ and nitrogen dioxide (NO_2_) emissions [11,12]. These changes may alter the toxicity of air pollutants, which in turn may lead to changes in the health effects of air pollution.

Previous studies have shown that air pollution may play an important role in the causes of AR [13,14,15]. However, to date, no studies have elucidated the temporal changes in the associations between ambient air pollution and daily outpatient visits for AR in China over a long-term scale. Therefore, the purpose of this study was to examine whether there is a change in the short-term effects of six air pollutants on AR outpatient visits in Beijing, China, over a seven-year period from 2014 to 2020. Furthermore, the effect values and exposure-response curves for each specific period were estimated.

## 2. Methods

### 2.1. Study Area and Data Collection

The daily data on AR outpatient visits from 1 January 2014 to 31 December 2020 were collected from the Chinese People’s Liberation Army (PLA) Strategic Support Force Characteristic Medical Center, one general hospital (the area of 100,000 square meters) in Beijing. It is a tertiary A-level hospital, providing 24 h accident and emergency services for residents. The data contains information on patient ID, age, sex and visit date. The cases of AR were coded according to the 10th edition of the International Classification of Diseases (ICD-10, J30.401). The cases living in Beijing and visiting for an AR occurrence or exacerbation were selected for subsequent analysis. We excluded people who visited a doctor only for medicine prescriptions. The data were grouped according to sex (male and female) and age (18–45 years, 46–65 years, and >65 years). All of the procedures in this study were approved by the Science and Technology Ethics Review Committee of the University of Science and Technology Beijing.

We collected hourly data of the air pollutants (PM_10_, PM_2.5_, SO_2_, NO_2_, carbon monoxide (CO) and ozone (O_3_)) during 2014–2020 from the National Urban Air Quality Publishing Platform (http://106.37.208.233:20035/ (accessed on 15 March 2021)). For each pollutant except O_3_, there are at least 20 h of hourly data per day to determine the daily mean concentration. The maximum daily 8 h moving average concentration for O_3_ was calculated using hourly data for at least 6 h. The city-level average concentrations were calculated based on 35 routine monitoring stations within the city. Further adjustment of the meteorological variables to control for potential confounding effects. The daily mean temperature (°C) and relative humidity (%) were obtained from the National Meteorological Information Center (http://data.cma.cn/site/index.html (accessed on 15 March 2021)). The spatial distribution of the environmental monitoring stations, the weather station and the hospital in Beijing is shown in Figure S1.

### 2.2. Statistical Analysis

The daily outpatient visits for AR, pollutant concentrations and meteorological factors during the study period were presented in means and standard deviation (SD). In addition, the parameters of 25% quartile, 50% quartile, 75% quartile, the interquartile range (IQR) and the minimum and maximum were described.

Since the daily outpatient visits approximately follow a quasi-Poisson distribution, a generalized additive model was carried out to analyze the associations between the daily outpatient visits for AR and each air pollutant [16,17]. The number of outpatient visits served as the dependent variable, and the fitting equation was obtained using the quasi-Poisson copula function. We defined the current day air pollution exposure as lag0 and examined the lagged day from lag1 to lag5. We also examined the accumulated effects of the multi-day lags using the moving average for the current day and previous 1–5 days (from lag01 to lag05). Several covariates, including natural splines, were adjusted based on previous studies [9,18]: (1) the natural spline smooth function of the calendar day with 7 degrees of freedom (df) per year to control for the underlying time trends; (2) an indicator variable for the day of the week to account for within week variations; and (3) two separate natural splines with 3 df for the mean temperature and 3 df for the mean relative humidity to exclude potential non-linear effects of the weather conditions. The choice of the most appropriate df for the weather variables was based on previous studies [19,20] and also on Akaike’s information criterion (AIC) [21] (Table S1). The main model is as follows:

log[E(Y_t_) = intercept + β X_t_ + s (time, df = 7 × year) + s (temperature, df = 3) + s (relative humidity, df = 3) + DOW

where Y_t_ represents the number of outpatient visits for AR on day t; X_t_ is the city-average concentration of a given air pollutant at day t; β is the regression coefficient; s indicates the natural spline function; df is the degree of freedom; time indicates long-term trends and seasonality using calendar time (days); and DOW is an indicator variable meaning “day of the week”.

To explore the temporal variation in the impact of air pollution on the outpatient visits for AR over the study period 2014–2020, we divided the study time into three periods, period 1 (2014–2015), period 2 (2016–2017) and period 3 (2018–2020). This is mainly in consideration of the implementation of the Law of the People’s Republic of China on Prevention and Control of Air Pollution on 1 January 2016 and the release of the Blue Sky Protection Campaign in 2018. We included an interaction term for the period variable and the air pollutant in the main model to validate changes in risk estimates over the specific period. We used *p* < 0.05 to assess the significant change of this linear interaction effect [7,8].

In line with previous studies [9,22], the analysis was also stratified by a cool season (October to March), a warm season (April to September), a pollen season (April, May, August, and September) and a non-pollen season (the out of pollen season months). We also alternated the degrees of freedom of the calendar time from 4 to 10 to assess the robustness of the effect estimates.

In the sensitivity analysis, additional period-specific analyses were conducted at overlapping 2 year intervals to assess the continuous changes in health risks associated with air pollutants over the study period [6,8]. Specifically, we assessed the effects of air pollution on daily outpatient visits for AR during the periods 2014–2015, 2015–2016, 2016–2017 and so on, up to 2019–2020. The Mann–Kendall statistical test was used to evaluate the probable temporal trends in the associations between air pollution and outpatient visits [7,23]. We further considered the offset term for the logarithmic scale of the annual population [24], the season and the three-day moving average temperature in the model, and performed a sensitivity analysis.

Finally, the smoothing function of the generalized additive model was employed to graphically describe the probable variations in period-specific associations. During three specific periods, we investigated the non-linear exposure-response relationships between air pollution and AR daily visits.

All statistical analyses were conducted in R (version 3.6.3, R Foundation for Statistical Computing, Vienna, Austria). The statistical test was two-sided, and the associations of *p* < 0.05 were considered statistically significant. The effects are presented as the relative risk (RR) and its 95% confidence intervals (CIs) in the daily AR outpatient visits for each interquartile range (IQR) increase in pollutant concentrations.

## 3. Results

Table 1 presents the descriptive statistics of hospital outpatient visits for AR, air pollutant concentrations and meteorological measures. A total of 68,861 AR outpatient visits were recorded (mean 27 per day), with 63.0% of patients being males and 59.3% of people aged 18–45 years.

**Figure 1:**
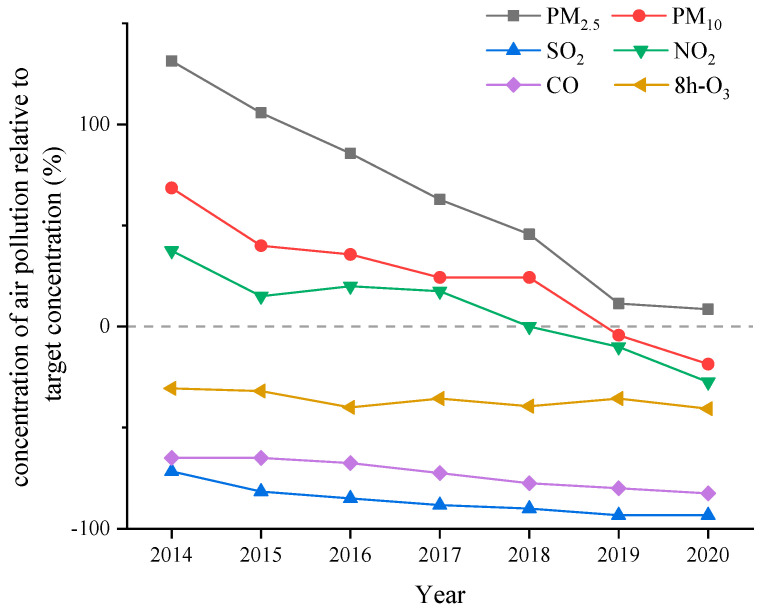
Annual average concentrations of six air pollutants in Beijing in 2014–2020, as percentages of change compared with the Chinese national ambient air quality standard. Note: The dashed line denotes the Chinese national ambient air quality standard. Values are the percentage increase or decrease of each concentration relative to the standard value (0%). The standards and the measured data are all the daily average.

Throughout the entire study period, the daily mean concentrations of PM_2.5_, PM_10_, SO_2_, NO_2_, CO and O_3_ were 60.6 μg/m^3^, 86.0 μg/m^3^, 9.2 μg/m^3^, 43.2 μg/m^3^, 1.1 mg/m^3^ and 96.9 μg/m^3^, respectively. The daily average temperature was 15.7 ℃, and the relative humidity was 48.7%. A substantial decline in the annual concentration of ambient air pollution was noted over the study period from 2014 to 2020 (Figure 1). The annual concentrations of ambient PM_2.5_ ranged between 81 μg/m^3^ and 38 μg/m^3^ from 2014 to 2020, which were higher than the NAAQS of 35 μg/m^3^ for PM_2.5_. The annual concentrations of ambient PM_10_ varied between 118 μg/m^3^ and 57 μg/m^3^ and NO_2_ varied between 55 μg/m^3^ and 29 μg/m^3^. The annual concentrations of 2019 and 2020 were below the NAAQS of 70 μg/m^3^ for PM_10_ and 40 μg/m^3^ for NO_2_. The annual concentrations of SO_2_ varied between 17 μg/m^3^ and 4 μg/m^3^ and O_3_ μg/m^3^ varied between 111 μg/m^3^ and 95 μg/m^3^. The annual average concentrations of SO_2_, O_3_ and CO during the entire study period were below the NAAQS of 60 μg/m^3^ for SO_2_, 4 mg/m^3^ for CO and 160 μg/m^3^ for O_3_.

**Figure 2:**
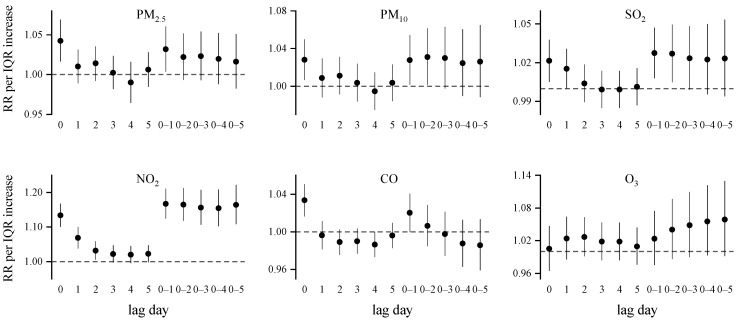
Relative risks (RRs) of outpatient visits for AR associated with an IQR increase in the air pollutants at different lag days during 2014–2020. IQR: PM_2.5_, 55 μg/m^3^; PM_10_, 70 μg/m^3^; SO_2_: 7 μg/m^3^; NO_2_: 27 μg/m^3^; CO: 0.5 mg/m^3^; O_3_: 86 μg/m^3^.

Figure 2 shows the lag distribution of the associations between air pollutants and outpatient visits for AR. The strongest effects were found at lag0 for PM_2.5_, and CO, at lag01 for SO_2_ and NO, and at lag02 for PM_10_. No significant effect was found for O3 neither in the single lag days nor in the cumulative lag days. Specifically, the RR (95%CI) of each IQR increase in PM_2.5_, PM_10_, SO_2_ and NO_2,_ and the CO concentration corresponded to 1.042 (1.016–1.069), 1.031 (1.002–1.061), 1.027 (1.008–1.047), 1.167 (1.125–1.211) and 1.033 (1.016–1.050) on AR visits, respectively. We also report the estimated effects per an increase of 10 μg/m^3^ to demonstrate the comparable results for the same increase in the pollutant (Table S2). In the sensitivity analysis, the associations of the air pollutants with AR outpatient visits remained stable when changing the degree of freedom (df) of time (4–10 per year) (Figure S2), replacing the pollutant concentrations with the nearest monitoring site (Figure S3) and adding the population (Table S3), season and three-day moving average temperature into the model (Table S4).

Table 2 shows the season-stratified analysis of the short-term effects of air pollution. The significant higher effects of PM_2.5_, SO_2_ and CO were found in warm seasons. A significant effect of O_3_ was only found in the pollen season. The associations between NO_2_ and AR visits were significant in different season groups, of which the higher effects occurred in cool and non-pollen seasons. The associations in most of the two-pollutant models did not change substantially, except that the associations with PM_10_ and SO_2_ became insignificant when further adjusted for NO_2_ or CO (Table S5).

Table 3 presents the estimated effects associated with the increased IQR of the air pollutant concentration at lag0 over three specific periods. Although the changes in the effect estimates were not significant for most of the pollutants, it was possible to find the patterns in different pollutants over time. For PM_2.5_, a significant effect of air pollution was only observed in period 3, while no such association was found in the previous periods. Differently, significant effects were only observed in period 1 for PM_10_ and CO. For SO_2_, the highest effect was observed in period 3, while the lowest in period 2. Over the study period, the associations between NO_2_ and AR visits were generally steady, with the highest effect reported in period 3 and the lowest reported in period 1. The temporal variations of the air pollution effects were also explored at lag01 for SO_2_ and NO_2_, and at lag02 for PM_10_, which considered that the exposure window of the largest effect occurred (Table S6). However, time changes remain consistent, indicating that the findings of this study were relatively stable throughout different lag days.

**Figure 3:**
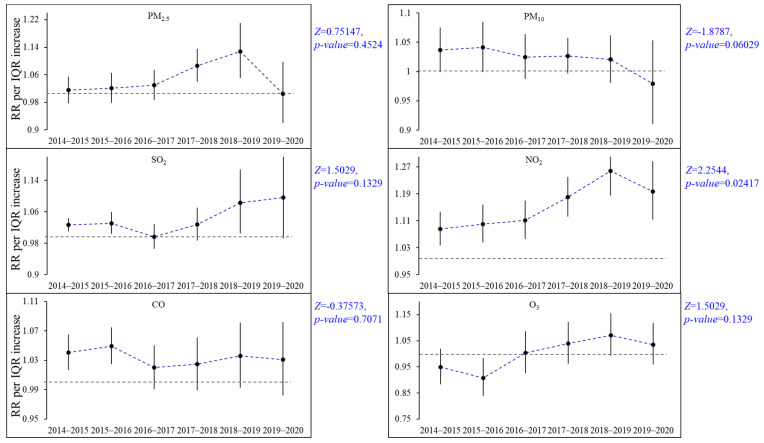
Relative risks (RRs) of AR visits for an IQR increase of air pollutants at lag0 in the period-specific analysis of overlapping 2-year intervals. The dotted curve lines indicate the trend of the mean risk estimate. Note: *Z* and *p*-values indicate the results of the Mann–Kendall test.

Figure 3 presents the period-specific assessment of the overlapping 2-year intervals for each air pollutant. Some increase in effect was observed in PM_2.5_ from 2014 to 2019, especially from 2017 to 2019. The impact of PM_10_ and CO showed a decreasing trend and the specific-period effects shifted from significant to insignificant. The estimated effect of SO_2_ showed the lowest value in 2016–2017 and the overall showed an increasing trend. The effect estimates of NO_2_ remained significant for each specific period and showed a significant increase in effects over time (Mann–Kendall test, Z = 2.2544, *p* = 0.02417). O_3_ showed a significant positive effect in 2015–2016 and a negative but insignificant effect in the subsequent period. Further studies are needed on the effect of O_3_ on respiratory diseases.

**Figure 4:**
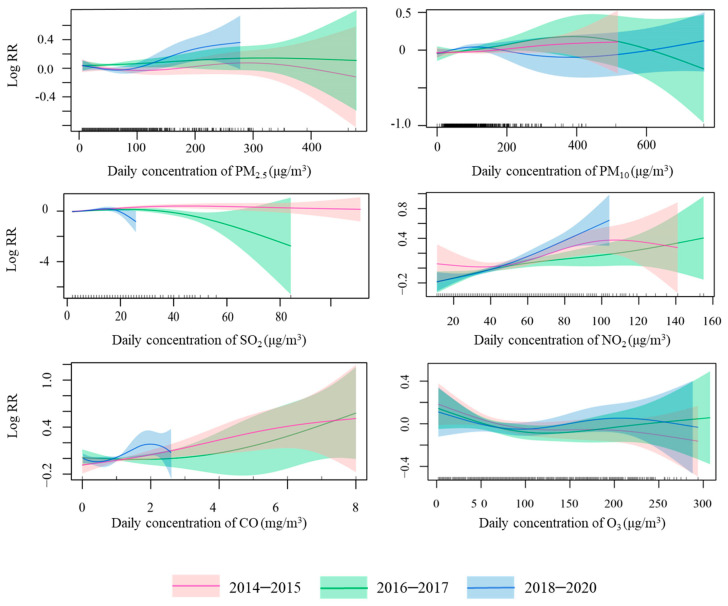
The concentration-response relationship curves of air pollutants versus AR visits for specific periods. Note: The *X*-axis represents the moving average concentration at lag0. The *Y*-axis represents the log relative risk (RR). The lines show the mean estimates, and the ribbons show the 95% confidence intervals.

Figure 4 presents the concentration-response curves for the associations between air pollutants (at lag0) and AR visits for three specific periods. The curves for PM_2.5_ for periods 1 and 2 overlapped well, however the curve for period 3 was barely overlapped by both curves. At concentrations around 100–200 μg/m^3^, the three curves were approximately linear, with the highest mean estimate observed in period 3 and the lowest in period 1. The three curves for PM_10_ were approximately linear at low concentrations (0–100 μg/m^3^) and overlapped each other. However, due to the sparse data at the high concentrations, caution should be exercised in interpreting the relationship curves. For SO_2_, the three curves were approximately linear at low concentrations (0–20 μg/m^3^) with good overlap, while at higher concentrations (>20 μg/m^3^) there was almost no overlap. For NO_2_, the three curves were approximately linear at relatively low concentrations (40–100 μg/m^3^), but exhibited different patterns at higher concentrations (>100 μg/m^3^). The mean estimate line was steeper and had a greater slope in period 3 than in period 1 and period 2. For CO, the three curves showed different shapes for specific periods. However, the concentration data were sparse and therefore need to be interpreted with caution. For O_3_, the three curves overlapped each other and had almost the same shape.

## 4. Discussion

In this study, we examined the changes in the short-term effects of air pollutants on AR visits from 2014 to 2020 in Beijing, China. We observed significant and positive relationships between the air pollutants concentration and AR visits. To our knowledge, this is the first study to investigate the temporal trends in the effect of air pollution on the outpatient visits for AR in such a time-series analysis. Between 2014 and 2020, the annual average concentrations decreased for ambient air pollutants. However, air pollution associated with AR risks continued to be significant during the study period. Furthermore, despite the remarkable reductions in ambient air pollutant concentrations, AR outpatient risks associated with PM_2.5_, SO_2_ and NO_2_ remained significant in recent years, even with a significant increase in NO_2_ over time. This result was further elucidated by the exposure-response relationship curves in specific periods.

During our study period, substantial declines in the annual mean concentrations of air pollutants were found from 2014 to 2020, especially for PM_2.5_, PM_10_ and NO_2_. The concentrations presented a relatively high level during period 1 and period 2. Following 2018, the emissions of pollutants decreased continuously due to the implementation of the control measures in the area. Except for PM_2.5_, the concentrations of pollutants were below the NAAQS in the late stage of period 3. Consistently, Maji et al. [25] reported that Beijing’s air quality has seen a dramatic improvement over 2014–2018, which can be attributable to the enforcement of the Air Pollution Prevention and Control Action Plan (APPCAP) regulation. One study showed that air pollution control measures implemented in China reduced the PM_2.5_ pollution in Beijing by an average of 11% during 2008–2019 [26]. The air quality improvement achievable under the Clean Air Action Plan (CAAP) in Beijing were evaluated, with the largest reduction of 87% in SO_2_ emissions, which was associated with strong coal combustion controls [27].

Our time-series study supported the comprehensive epidemiological evidence regarding the short-term effects of ambient air pollution on AR outpatient visits. In the present study, the significant effects on AR visits were found for all pollutants except O_3_. These associations were generally similar to those reported in previous studies [28,29,30,31]. Consistently, we found a comparatively strong association between NO_2_ and AR visits, with an RR of 1.167 (1.125–1.211). According to several studies, the NO_2_ levels are a strong indicator of negative health effects [32]. The strong associations between NO_2_ and AR have been observed in several systematic reviews and meta-analyses [13,33]. The exact mechanisms that underlie the associations between air pollutants and AR are not well established. NO_2_ may damage the nasal mucosa, impair the clearance of mucocele and cause the production of eosinophils, which cause airway hypersensitivity reactions [34,35]. Moreover, the observed differences between the air pollutants and AR might be due to the homogeneity of the materials adsorbed on pollutants, and this homogeneity will vary across the regions [36].

Previous studies have typically reported constant risk estimates per unit increase in air pollutants over the study period. However, few studies have looked into whether the risks of air pollution vary over time, particularly in developing countries [5,6,8]. Therefore, the temporal variability of air pollutants effects needs further study, as air pollution mixtures may vary over time as a result of policy implementation, changes in weather patterns and emission sources [37]. Additionally, biomass fuels and vehicle emissions play a role in the formation of secondary pollutants. Although pollutant concentrations have fallen, secondary products of air pollution may change, causing health effects [38]. Scientific knowledge on the temporal changes in air pollution impacts may be needed to assess whether current policy actions are sufficient to effectively control ambient air pollution to reduce public health concerns.

Several previous studies have explored the temporal variation in the short-term effects of air pollution, but the findings remained inconsistent. A study conducted in Shanghai over 12 years found that the short-term effects on emergency department visits for NO_2_ remained stable and even increased for PM_10_ [8]. Despite non-homogenous decreasing trends in annual concentrations during the study period, the effects of PM_10_ on cardiovascular and respiratory admissions remained significant and even showed an increasing trend for cardiovascular admissions [5]. Despite drastic reductions in annual levels, the risk of cardiovascular and respiratory mortality associated with PM_2.5_ remained significant and even increasing [7]. However, a study conducted in Italy reported that the effect estimates for a fixed increment in each exposure were generally consistent, even the mean concentrations of air pollutants have decreased over the last two decades [6]. A study conducted in Guangzhou over more than a decade reported that the attributable fraction of NO_2_ on total mortality decreased from 1.38% to 0.43%, while the average annual concentrations decreased during 2006–2016 [39]. In contrast, one study reported a trend toward the increased risk of daily nonaccidental deaths from NO_2_, despite the decreasing concentration of NO_2_ [40].

In the present study, the increased risk of AR associated with PM_2.5_ observed in the region during the recent period might be explained by the changes in the composition of the PM mixture over time. The exposure-response relationship curves for period 1 and period 2 almost coincided, but there was little overlap with the curves for period 3. This suggests that the exposure-response association in period 3 is distinct from that of period 1 and period 2. Therefore, we speculate that the period-specific variations might be due to the changes in the PM_2.5_ composition and toxicity [41]. Nitrated aromatic compounds (NACs), a group of pollutants bound to particles, attracted widespread attention owing to their remarkable toxicity and allergenicity [42]. Shi et al. reported that the annual levels of primary nitrated polycyclic aromatic hydrocarbons (NPAHs) in Beijing decreased by 46.3–54.8% from 2012–2013 to 2016–2018, but the secondary species did not change significantly [43]. One study separated and identified six sources of PM_2.5_ in Beijing and found that the BaPeq toxicity, due to coal combustion, may pose both long-term and short-term health risks [44]. The implementation of air pollution control measures could be effective in reducing industrial sources, but might be less effective for some sources of PM_2.5_. Thus, despite the reduction of the PM_2.5_ concentrations during the study period, the estimated effect on AR visits has increased in recent years.

This study found a significant association between SO_2_ and AR in period 1 and period 3, but not in period 2. In developing countries, SO_2_ remains a major component of air pollution. Despite decreasing concentrations, studies have found an increased risk of mortality from SO_2_ [6,45]. Unlike PM_10_, SO_2_ exposure is not easily intervened by adaptive behaviors such as wearing a mask or staying indoors. In addition, we found that SO_2_ impact estimates decreased or even shifted to a positive effect after adjusting for NO_2_ throughout the period of this study. However, adjusting for SO_2_ did not change the impact of NO_2_. NO_2_ reduced the effect of SO_2_ with AR, possibly because both pollutants are emitted from the same industrial or transportation source [46]. SO_2_ may act as a substitute for other toxic components of NO_2_. Han et al. reported that the influences of SO_2_ on deaths from respiratory diseases displayed downward trends with the decreasing pollution levels in Beijing [47]. However, one study found a significant relationship between the increased acute air pollution episodes and the increased hospitalizations for acute exacerbations of chronic respiratory diseases with ambient concentrations of SO_2_ decreased by 68% over 2013–2017 [9]. Various factors, such as demographic characteristics and spatial variations, may be modified to further investigate the health effect of SO_2_ in the future.

In the present study, the short-term effect of NO_2_ on AR outpatient visits remained stable and significant over time, and even a significant increasing trend was found, despite the non-homogenous decrease in the NO_2_ concentration. NO_2_ is known to be a good indicator of traffic pollutants, such as polycyclic aromatic hydrocarbons and volatile organic compounds, which have negative health effects [48,49]. One possible explanation for the study findings is that the composition of the traffic emissions may have changed or even the overall toxicity of the traffic-sources of air pollutants has become stronger [48]. Although we found trends of an increased risk for PM_2.5_, SO_2_ and NO_2_, the changes in the energy structure, fuel composition and the regional demographics may have contributed to the variability around the central tendency for the mean risk estimates over time. We considered the population factor in the sensitivity analysis, while questions about energy structure and fuel composition need to be further explored in future studies.

The present study has some limitations. Firstly, the daily average concentrations of pollutants were obtained from air monitoring stations, and we assume that the same level of exposure to air pollutants was shared by residents. The data might not be a good representation of exposure for the total population, resulting in exposure assessment errors. Furthermore, considering the limited availability of monitoring stations, the exposure assessment may be biased. To assess the air pollutant concentrations in unmonitored areas, a spatial interpolation to quantify individual outdoor exposures is an effective method. Secondly, this study was conducted in only one city, and the results might not apply to any other city because the components that influence the overall toxicity of pollutants may differ. Thirdly, the lack of this information limits us to evaluate the composition variation of air pollutants and their potential toxicity on AR visits. In addition, information about indoor allergens such as house dust mite, cockroach and furry pet allergens in subjects was not available, which may also vary over time. Additional studies are required to further investigate this issue. However, the strength of this study is noteworthy because temporal variations of the short-term effects of air pollution on AR outpatient visits in Beijing, China were observed. The effects of PM_2.5_ and SO_2_ were significant in the last period and the effect of NO_2_ showed significant and stable effects over the entire period, even though the concentrations continued to decline. This finding could lead to a better understanding of the sources of pollutants that are the most economical factors to control in terms of reducing health effects. The results of this study can be taken as scientific evidence to guide provincial policy decisions.

## 5. Conclusions

Our study suggested that short-term exposure to air pollution could significantly increase AR risk in Beijing. Despite the substantial reductions in the ambient concentrations, the short-term effects of PM_2.5_ and SO_2_ remained strong and that of NO_2_ even increased over time in Beijing. These findings suggest further investigation of the temporal changes in short-term effects of air pollution in China, and continued air pollution control efforts to reduce air pollution and protect the public in the future.

## Figures and Tables

**Table 1:** Descriptive statistics for AR visits, air pollutants and meteorological parameters in Beijing, China, 2014–2020.

	Period	Mean	SD	Min	Median	Max	IQR
Air pollutant concentration							
PM_2.5_ (μg/m^3^)	2014–2020	60.6	57.3	3.0	44.0	477.0	55.0
	2014–2015	82.3	70.5	5.0	62.0	477.0	80.0
	2016–2017	65.6	60.3	6.0	48.0	454.0	60.0
	2018–2020	42.7	35.7	3.0	34.0	233.0	38.0
PM_10_ (μg/m^3^)	2014–2020	86.0	67.8	7.0	69.0	831.0	70.0
	2014–2015	108.5	79.9	7.0	90.0	550.0	91.0
	2016–2017	91.7	71.4	7.0	76.0	764.0	73.0
	2018–2020	67.3	48.5	8.0	56.0	831.0	47.0
SO_2_ (μg/m^3^)	2014–2020	9.2	12.8	2.0	4.0	133.0	7.0
	2014–2015	16.7	19.6	2.0	9.0	9.0	17.0
	2016–2017	8.8	9.5	2.0	5.0	5.0	8.0
	2018–2020	4.5	3.1	2.0	3.0	3.0	3.0
NO_2_ (μg/m^3^)	2014–2020	43.2	22.4	5.0	38.0	155.0	27.0
	2014–2015	51.9	24.3	8.0	46.0	141.0	27.0
	2016–2017	47.1	22.7	11.0	42.0	155.0	25.0
	2018–2020	34.8	17.3	5.0	30.0	105.0	22.0
CO (mg/m^3^)	2014–2020	1.1	0.9	0.1	1.0	8.0	0.5
	2014–2015	1.4	1.0	0.1	1.0	8.0	1.0
	2016–2017	1.2	0.9	0.1	1.0	8.0	0.0
	2018–2020	0.8	0.5	0.1	1.0	3.0	0.7
8 h-O_3_ (μg/m^3^)	2014–2020	96.9	61.9	2.0	82.0	311.0	86.0
	2014–2015	99.4	65.2	2.0	87.0	294.0	94.0
	2016–2017	97.2	65.7	2.0	83.0	311.0	89.0
	2018–2020	95.1	56.8	2.0	80.0	283.0	76.5
Meteorological measures							
Temperature (°C)	2014–2020	15.7	9.9	−14.3	17.5	32.6	15.2
Relative humidity (%)	2014–2020	48.7	19.0	8.0	48.0	99.0	28.2
Outpatient hospital visits (n/day)							
Total	2014–2020	27	21	2	22	175	19
Male	2014–2020	17	14	3	13	120	13
Female	2014–2020	10	8	2	8	75	8
Age 18–45	2014–2020	16	15	1	12	123	12
Age 46–65	2014–2020	6	5	2	5	31	5
Age > 65	2014–2020	2	2	3	2	16	3
Warm season (4–9)	2014–2020	31	22	2	26	175	24
Cool season (10-3)	2014–2020	23	20	2	19	160	15
Pollen season (4–5, 8–9)	2014–2020	39	23	3	34	175	27
Non-pollen season (others)	2014–2020	21	18	1	18	160	13

Note: Values of PM_2.5_, PM_10_, SO_2_, CO and NO_2_ were the 24 h mean concentration; values of O_3_ were computed using the 8 h mean concentrations; values of meteorological factors were the daily average. Abbreviations: SD, standard deviation; Min, minimum; Max, maximum; IQR: interquartile range.

**Table 2:** Relative risks (RRs) of outpatient visits for allergic rhinitis associated with an IQR increase in the air pollutants for different seasons ^a^.

	Warm Season	Cool Season	Pollen Season	Non-Pollen Season
PM_2.5_	1.044 (1.009–1.081) *	1.027 (0.999–1.055)	1.032 (0.992–1.074)	1.010 (0.985–1.035)
PM_10_	1.020 (0.992–1.048)	1.009 (0.986–1.031)	1.014 (0.985–1.045)	1.012 (0.991–1.033)
SO_2_	1.030 (1.001–1.059) *	1.011 (0.996–1.025)	1.027 (0.995–1.060)	1.009 (0.995–1.023)
NO_2_	1.079 (1.031–1.129) *	1.091 (1.058–1.125) *	1.073 (1.020–1.128) *	1.086 (1.057–1.117) *
CO	1.027 (1.005–1.050) *	1.001 (0.985–1.018)	1.019 (0.991–1.047)	1.002 (0.987–1.017)
O_3_	1.035 (0.998–1.073)	0.950 (0.870–1.037)	1.050 (1.006–1.096) *	0.974 (0.923–1.028)

^a^ the estimate effects were evaluated at lag 0 for the different pollutants.* *p* value < 0.05.

**Table 3:** Relative risks (RRs) and 95% confidence interval of outpatient visits for AR for an IQR increase of air pollutants at lag0 during specific periods.

Pollutant	Period 1	Period 2	Period 3	*p* Value ^a^
PM_2.5_	1.015 (0.978, 1.054)	1.030 (0.987, 1.075)	1.069 (1.005, 1.135)	0.771
PM_10_	1.037 (1.001, 1.075)	1.025 (0.987, 1.063)	1.008 (0.971,1.046)	0.123
SO_2_	1.027 (1.009, 1.044)	0.997 (0.966, 1.028)	1.074 (1.003, 1.149)	0.725
NO_2_	1.086 (1.037, 1.137)	1.111 (1.055, 1.170)	1.214 (1.149, 1.282)	0.051
CO	1.041 (1.017, 1.065)	1.020 (0.991, 1.050)	1.027 (0.990, 1.066)	0.112
O_3_	0.949 (0.883, 1.019)	1.003 (0.927, 1.087)	1.040 (0.975, 1.105)	0.353

^a^*p* value of the linear interaction term between the air pollutants and time periods.

## Data Availability

Not applicable.

## Supplementary Materials

The following supporting information can be downloaded at: https://www.mdpi.com/article/10.3390/ijerph191912529/s1, Figure S1: Spatial distribution of the environmental monitoring stations, the weather station and the hospital in Beijing; Figure S2: Relative risks (RRs) of outpatient visits for AR associated with an IQR increase in the air pollutants using different degrees of freedom per year; Figure S3: Relative risks (RRs) of outpatient visits for AR associated with a 10 μg/m^3^ (CO, 1 mg/m^3^) increase in the air pollutants at different lag days during 2014–2020. The data of air pollutants was obtained from the monitoring station closest to the hospital; Table S1: AIC values for changing the degrees of freedom of the meteorological factors in the model; Table S2: Relative risks (RRs) of outpatient visits for AR associated with a 10 μg/m^3^ (CO, 1 mg/m^3^) increase in the air pollutants at different lag days during 2014–2020; Table S3: Relative risks (RRs) of outpatient visits for AR associated with a 10 μg/m^3^ (CO, 1 mg/m^3^) increase in the air pollutants through the original model and the model plus demographic factors; Table S4: Relative risks (RRs) of outpatient visits for AR associated with a 10 μg/m^3^ (CO, 1 mg/m^3^) increase in the air pollutants through the original model and the model plus the season and the three-day moving average temperature; Table S5: Relative risks (RRs) of outpatient visits for AR associated with an IQR increase in the air pollutants at lag0 in the single-pollutant models and two-pollutant models; Table S6: Relative risks (RRs) of outpatient visits for AR for an IQR increase of SO_2_ and NO_2_ at lag01, and PM10 at lag02 during specific periods.
