# Supplementary material for: Has the Risk of Outpatient Visits for Allergic Rhinitis, Related to Short-Term Exposure to Air Pollution, Changed over the Past Years in Beijing, China?
Source: Int J Environ Res Public Health. 2022 Oct 1;19(19):12529. doi: 10.3390/ijerph191912529 (PMC9566797; doi:10.3390/ijerph191912529)
Supplement: Supplementary file 1 [file ijerph-19-12529-s001.zip › ijerph-1929737-supplementary.pdf]

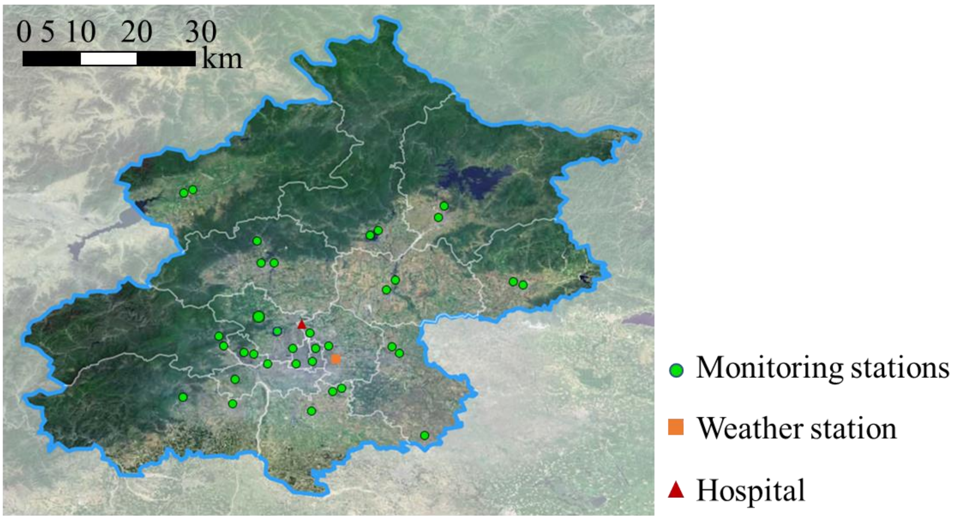

Figure S1 Spatial distribution of environmental monitoring stations, weather station and the hospital in Beijing.

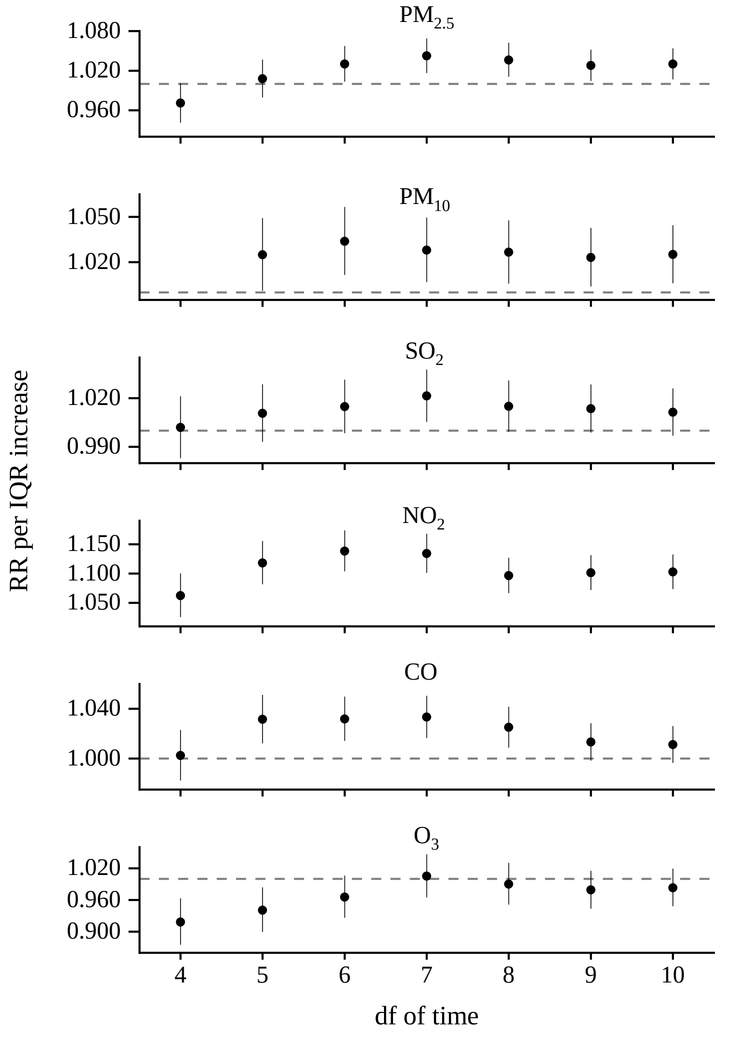

Figure S2 Relative risks (RRs) of outpatient visits for AR associated with an IQR increase in the air pollutants using different degree of freedom per year.

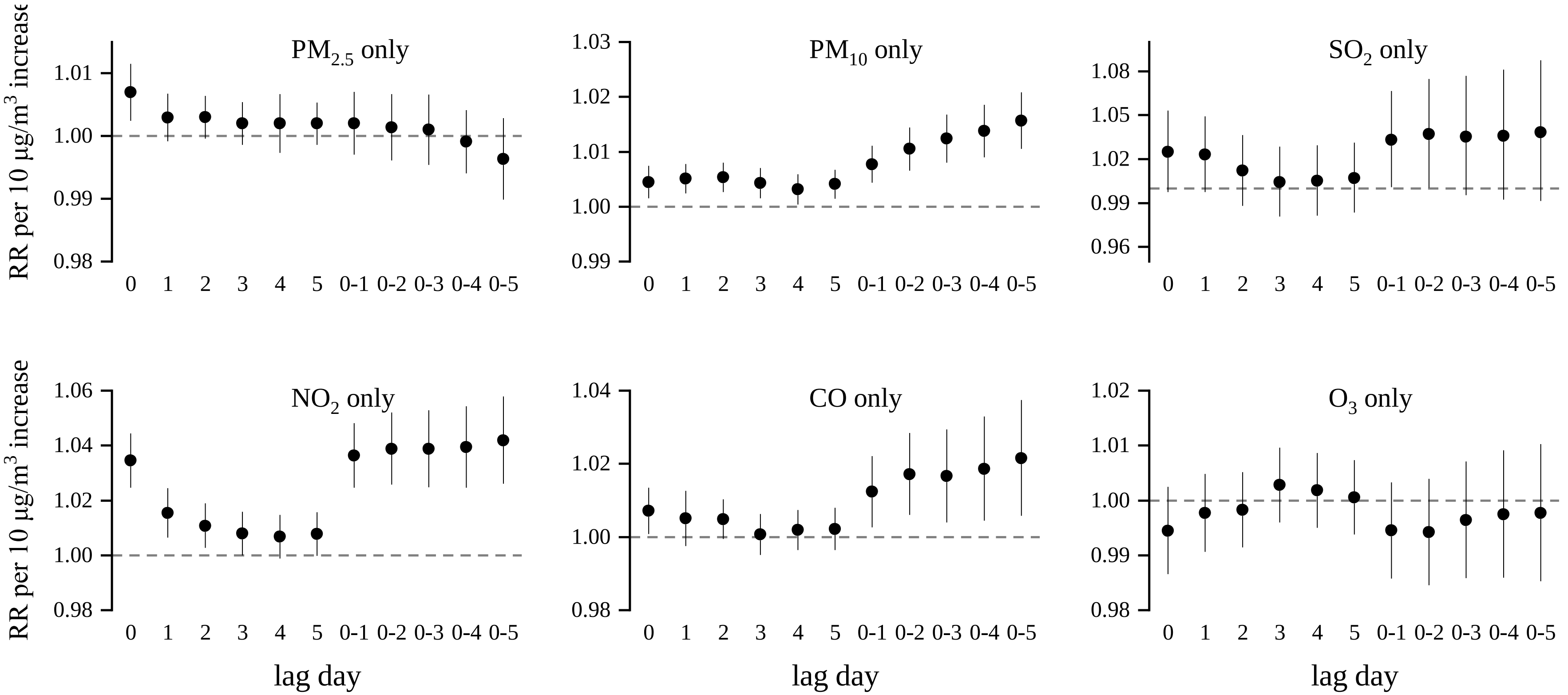

Figure S3 Relative risks (RRs) of outpatient visits for AR associated with a 10 μg/m^3 (CO, 1 mg/m^3) increase in the air pollutants at different lag days during 2014-2020. The data of air pollutants was obtained from the monitoring station closest to the hospital.

Table S1 AIC values for changing the degrees of freedom of meteorological factors in the model.

Pollutant models	Degree of freedom of temperature and relatively humidity	AIC [a]	Pollutant models	Degree of freedom of temperature and relatively humidity	AIC
$PM_{2.5}$	3	62.17	NO_2	3	61.95
	4	63.19		4	63.0
	5	65.13		5	64.97
	6	66.02		6	65.76
	7	66.9		7	66.61
PM_{10}	3	62.15	CO	3	62.13
	4	63.16		4	63.08
	5	65.08		5	64.85
	6	65.98		6	65.76
	7	66.87		7	66.64
SO_2	3	62.13	O_3	3	62.18
	4	63.14		4	63.21
	5	65.02		5	65.03
	6	65.9		6	65.96
	7	66.77		7	66.83

[a], Akaike's information criterion (AIC). The model that makes the AIC function smaller is considered to be the better model.

Table S2 Relative risks (RRs) of outpatient visits for AR associated with a 10 μg/m^3 (CO, 1 mg/m^3) increase in the air pollutants at different lag days during 2014-2020

Pollutant	Lag day	Per an increase of 10 μg/m^3 RR	95%CI	Pollutant	Lag day	Per an increase of 10 μg/m^3 RR	95%CI
$PM_{2.5}$	Lag 0	1.008	(1.003-1.012) *	NO_2	Lag 0	1.052	(1.039-1.064)
	Lag 1	1.002	(0.998-1.006)		Lag 1	1.027	(1.015-1.039)
	Lag 2	1.003	(0.999-1.006)		Lag 2	1.013	(1.002-1.023)
	Lag 3	1.000	(0.997-1.004)		Lag 3	1.009	(0.999-1.019)
	Lag 4	0.995	(0.991-0.998)		Lag 4	1.008	(0.998-1.018)
	Lag 5	1.001	(0.995-1.004)		Lag 5	1.009	(0.999-1.019)
	Lag 01	1.006	(1.001-1.012) *		Lag 01	1.064	(1.048-1.080)
	Lag 02	1.004	(0.999-1.010)		Lag 02	1.063	(1.046-1.080)
	Lag 03	1.004	(0.998-1.010)		Lag 03	1.060	(1.042-1.078)
	Lag 04	1.004	(0.997-1.010)		Lag 04	1.059	(1.040-1.079)
	Lag 05	1.003	(0.996-1.010)		Lag 05	1.063	(1.042-1.084)
PM_{10}	Lag 0	1.004	(1.001-1.007) *	CO	Lag 0	1.068	(1.033-1.104)
	Lag 1	1.001	(0.998-1.004)		Lag 1	0.992	(0.963-1.022)
	Lag 2	1.002	(0.999-1.004)		Lag 2	0.978	(0.952-1.005)
	Lag 3	1.000	(0.998-1.003)		Lag 3	0.980	(0.954-1.007)
	Lag 4	0.999	(0.996-1.002)		Lag 4	0.973	(0.947-0.999)
	Lag 5	1.000	(0.998-1.003)		Lag 5	0.992	(0.966-1.019)
	Lag 01	1.004	(1.000-1.007) *		Lag 01	1.040	(1.000-1.082)
	Lag 02	1.004	(1.000-1.008) *		Lag 02	1.013	(0.970-1.057)
	Lag 03	1.004	(1.000-1.009)		Lag 03	0.995	(0.950-1.043)
	Lag 04	1.003	(0.999-1.008)		Lag 04	0.975	(0.927-1.026)
	Lag 05	1.004	(0.998-1.009)		Lag 05	0.972	(0.920-1.026)
SO_2	Lag 0	1.036	(1.009-1.064) *	O_3	Lag 0	1.001	(0.995-1.006)
	Lag 1	1.026	(1.000-1.052)		Lag 1	1.003	(0.998-1.008)
	Lag 2	1.007	(0.983-1.031)		Lag 2	1.003	(0.999-1.008)
	Lag 3	0.999	(0.975-1.023)		Lag 3	1.002	(0.998-1.007)
	Lag 4	0.999	(0.975-1.023)		Lag 4	1.002	(0.998-1.007)
	Lag 5	1.002	(0.979-1.026)		Lag 5	1.001	(0.997-1.006)
	Lag 01	1.046	(1.013-1.080) *		Lag 01	1.003	(0.997-1.010)
	Lag 02	1.045	(1.008-1.083) *		Lag 02	1.005	(0.998-1.012)
	Lag 03	1.039	(0.999-1.082)		Lag 03	1.006	(0.999-1.014)
	Lag 04	1.037	(0.993-1.084)		Lag 04	1.007	(0.999-1.015)
	Lag 05	1.039	(0.990-1.090)		Lag 05	1.008	(0.999-1.016)

* P value < 0.05

Table S3 Relative risks (RRs) of outpatient visits for AR associated with a 10 μg/m^3 (CO, 1 mg/m^3) increase in the air pollutants through the original model and the model plus demographic factors.

Pollutant	Lag day	Original model	+offset (population)	Pollutant	Lag day	Original model	+offset (population)
$PM_{2.5}$	Lag 0	**1.008** *	**1.007** *	NO_2	Lag 0	**1.052** *	**1.053** *
	Lag 1	1.002	1.002		Lag 1	**1.027** *	**1.026** *
	Lag 2	1.003	1.002		Lag 2	1.013	**1.012** *
	Lag 3	1.000	1.001		Lag 3	1.009	1.009
	Lag 4	0.995	0.995		Lag 4	1.008	1.008
	Lag 5	0.998	0.996		Lag 5	1.009	1.009
	Lag 01	**1.001** *	**1.001** *		Lag 01	**1.064** *	**1.064** *
	Lag 02	1.004	1.004		Lag 02	**1.063** *	**1.063** *
	Lag 03	1.004	1.003		Lag 03	**1.060** *	**1.060** *
	Lag 04	1.004	1.004		Lag 04	**1.059** *	**1.060** *
	Lag 05	1.003	1.003		Lag 05	**1.063** *	**1.064** *
PM_{10}	Lag 0	**1.004** *	**1.004**	CO	Lag 0	**1.068** *	**1.066**
	Lag 1	1.001	1.001		Lag 1	0.992	0.989
	Lag 2	1.002	1.002		Lag 2	0.978	0.978
	Lag 3	1.000	1.001		Lag 3	0.980	0.982
	Lag 4	0.999	1.000		Lag 4	0.973	0.977
	Lag 5	1.000	1.001		Lag 5	0.992	0.995
	Lag 01	**1.004** *	1.003		Lag 01	1.040	1.036
	Lag 02	**1.004** *	**1.004**		Lag 02	1.013	1.009
	Lag 03	1.004	1.004		Lag 03	0.995	0.993
	Lag 04	1.003	1.004		Lag 04	0.975	0.976
	Lag 05	1.004	1.004		Lag 05	0.972	0.975
SO_2	Lag 0	**1.036** *	**1.036**	O_3	Lag 0	1.001	1.001
	Lag 1	**1.026** *	1.020		Lag 1	1.003	1.003
	Lag 2	1.007	1.006		Lag 2	1.003	1.004
	Lag 3	0.999	0.999		Lag 3	1.002	1.003
	Lag 4	0.999	0.999		Lag 4	1.002	1.003
	Lag 5	1.002	1.004		Lag 5	1.001	1.002
	Lag 01	**1.046** *	**1.041**		Lag 01	1.003	1.003
	Lag 02	**1.045** *	**1.041**		Lag 02	1.005	1.006
	Lag 03	1.039	1.036		Lag 03	1.006	1.007
	Lag 04	1.037	1.034		Lag 04	1.007	1.008
	Lag 05	1.039	1.037		Lag 05	1.008	1.009

* P value < 0.05

Table S4 Relative risks (RRs) of outpatient visits for AR associated with a 10 μg/m^3 (CO, 1 mg/m^3) increase in the air pollutants through the original model and the model plus the season and three-day moving average temperature.

Pollutant	Lag day	Original model	+season	+season+3-day moving average temperature
$PM_{2.5}$	Lag 0	**1.008** *	**1.007** *	**1.009** *
	Lag 1	1.002	1.002	1.001
	Lag 2	1.003	1.003	1.002
	Lag 3	1.000	1.001	1.002
	Lag 4	0.995	0.995	0.994
	Lag 5	0.998	0.998	0.997
	Lag 01	**1.001** *	**1.000** *	**1.001** *
	Lag 02	1.004	1.003	1.003
	Lag 03	1.004	1.004	1.004
	Lag 04	1.004	1.004	1.004
	Lag 05	1.003	1.003	1.003
PM_{10}	Lag 0	**1.004** *	**1.003** *	**1.003** *
	Lag 1	1.001	1.001	0.999
	Lag 2	1.002	1.002	0.998
	Lag 3	1.000	1.001	0.998
	Lag 4	0.999	1.000	0.999
	Lag 5	1.000	1.001	1.001
	Lag 01	**1.004** *	1.003	1.002
	Lag 02	**1.004** *	1.004	1.000
	Lag 03	1.004	1.004	0.998
	Lag 04	1.003	1.003	0.997
	Lag 05	1.004	1.004	0.998
SO_2	Lag 0	**1.036** *	**1.032** *	**1.031** *
	Lag 1	**1.026** *	1.020	1.022
	Lag 2	1.007	1.009	0.996
	Lag 3	0.999	1.001	0.987
	Lag 4	0.999	1.002	0.995
	Lag 5	1.002	1.006	1.005
	Lag 01	**1.046** *	**1.039** *	**1.040** *
	Lag 02	**1.045** *	**1.041** *	1.031
	Lag 03	1.039	1.037	1.018
	Lag 04	1.037	1.038	1.014
	Lag 05	1.039	1.042	1.018
NO_2	Lag 0	**1.052** *	**1.051** *	**1.058** *
	Lag 1	**1.027** *	**1.025** *	**1.029** *
	Lag 2	1.013	**1.012** *	1.002
	Lag 3	1.009	1.008	0.994
	Lag 4	1.008	1.007	1.000
	Lag 5	1.009	1.009	1.005
	Lag 01	**1.064** *	**1.062** *	**1.071** *
	Lag 02	**1.063** *	**1.061** *	**1.057** *
	Lag 03	**1.060** *	**1.058** *	**1.045** *
	Lag 04	**1.059** *	**1.057** *	**1.040** *

Pollutant	Lag day	Original model	+season	+season+3-day moving average temperature
	Lag 05	**1.063** *	**1.060** *	**1.043** *
CO	Lag 0	**1.068** *	**1.059** *	**1.069** *
	Lag 1	0.992	0.989	0.994
	Lag 2	0.978	0.979	0.963
	Lag 3	0.980	0.981	0.962
	Lag 4	0.973	0.975	0.966
	Lag 5	0.992	0.995	0.993
	Lag 01	1.040	1.032	**1.043** *
	Lag 02	1.013	1.007	1.001
	Lag 03	0.995	0.991	0.972
	Lag 04	0.975	0.972	0.949
	Lag 05	0.972	0.971	0.947
O_3	Lag 0	1.001	1.001	1.002
	Lag 1	1.003	1.003	0.996
	Lag 2	1.003	1.004	0.995
	Lag 3	1.002	1.002	0.998
	Lag 4	1.002	1.002	1.000
	Lag 5	1.001	1.002	1.001
	Lag 01	1.003	1.003	0.999
	Lag 02	1.005	1.005	0.995
	Lag 03	1.006	1.006	0.994
	Lag 04	1.007	1.007	0.994
	Lag 05	1.008	1.008	0.995

* P value < 0.05

Table S5 Relative risks (RRs) of outpatient visits for AR associated with an IQR increase in the air pollutants at lag0 in single-pollutant models and two-pollutant models.

Two-pollutant models		Total RR	Total 95%CI	Two-pollutant models		Total RR	Total 95%CI
$PM_{2.5}$	-	1.042	1.016-1.069*	NO_2	-	1.167	1.125-1.211*
	$+NO_2$	0.954	0.922-1.002		$+PM_{2.5}$	1.194	1.146-1.244*
	$+SO_2$	1.034	1.003-1.065*		$+SO_2$	1.174	1.046-1.076*
	+CO	1.021	0.990-1.053		+CO	1.152	1.110-1.196*
	$+O_3$	1.043	1.017-1.069*		$+O_3$	1.135	1.102-1.169*
PM_{10}	-	1.028	1.007-1.050*	CO	-	1.033	1.016-1.050*
	$+SO_2$	1.020	0.997-1.044		$+PM_{2.5}$	1.025	1.005-1.045*
	$+NO_2$	0.983	0.959-1.008		$+SO_2$	1.028	1.009-1.048*
	+CO	1.014	0.991-1.038		$+NO_2$	0.981	0.961-1.002
	$+O_3$	1.028	1.007-1.050*		$+O_3$	1.033	1.017-1.051*
SO_2	-	1.027	1.008-1.047*	O_3	-	1.005	0.965-1.047
	$+PM_{2.5}$	1.012	0.991-1.035		$+PM_{2.5}$	1.001	0.955-1.050
	$+NO_2$	0.980	0.961-1.002		$+SO_2$	1.012	0.965-1.060
	+CO	1.008	0.991-1.027		$+NO_2$	1.025	0.978-1.073
	$+O_3$	1.021	1.005-1.038*		+CO	1.013	0.966-1.061

* P value < 0.05

Table S6 Relative risks (RRs) of outpatient visits for AR for IQR increase of SO_2 and NO_2 at lag01, and PM_{10} at lag02 during specific periods.

Lag day	Pollutant	Entire period	Period 1	Period 2	Period 3	*P* value
At lag01 [a]	SO_2	1.027 (1.008, 1.047) *	1.263 (1.052, 1.517) *	1.041 (0.733, 1.477)	2.374 (1.068, 5.278) *	0.99421
	NO_2	1.167 (1.125, 1.211) *	1.180 (1.043, 1.334) *	1.330 (1.159, 1.527) *	1.768 (1.512, 2.067) *	0.01409
At lag02 [b]	PM_{10}	1.031 (1.002, 1.061) *	1.041 (1.001, 1.082) *	0.999 (0.960, 1.039)	1.008 (0.972, 1.044)	0.75829

[a], the largest effect estimates of SO_2 and NO_2 occurred at lag 01; [b], the largest effect estimates of PM_{10} occurred at lag 02.

* P value < 0.05
